# Reciprocal crosstalk between endometrial carcinoma and mesenchymal stem cells via transforming growth factor-β/transforming growth factor receptor and C–X–C motif chemokine ligand 12/C–X–C chemokine receptor type 4 aggravates malignant phenotypes

**DOI:** 10.18632/oncotarget.23212

**Published:** 2017-12-14

**Authors:** Dah-Ching Ding, Tang-Yuan Chu, Hwan-Wun Liu

**Affiliations:** ^1^ Department of Obstetrics and Gynecology, Buddhist Tzu-Chi General Hospital, Hualien, Taiwan; ^2^ Institute of Medical Sciences, Tzu Chi University; Hualien, Taiwan; ^3^ Cervical Cancer Prevention Center, Department of Research, Buddhist Tzu Chi General Hospital, Hualien, Taiwan; ^4^ Department of Occupational Medicine, Buddhist Tzu Chi General Hospital, Hualien, Taiwan

**Keywords:** endometrial cancer, mesenchymal stem cells, transforming growth factor-ß1, C–X–C motif chemokine ligand 12, C–X–C chemokine receptor type 4

## Abstract

Designated for cyclic shedding, the endometrial stroma is rich in endometrial mesenchymal stem cells (EMSCs) and may play an important role in the development of endometrial carcinoma (EC). This study characterized the crosstalk of EC cells with EMSCs and the resultant effects on malignant phenotypes. The cultured EMSCs expressed CD73, CD90, and CD105, but not CD14, CD19, CD34, CD45, or human leukocyte antigen—antigen D related markers. These EMSCs also showed osteogenic, adipogenic, and chondrogenic differentiation ability. Transforming growth factor (TGF)-β1 and C–X–C motif chemokine ligand 12 (CXCL12) secretion or expression were reciprocally enhanced in EC cells and EMSCs, as well as in their tissues. By acting on the receptors expressed in their mutual target cells, the interaction between TGF-β and CXCL12 results in the enhanced migration, invasion, tumorigenesis, and epithelial–mesenchymal transition of EC cells, which can be blocked by neutralizing the antibody of either CXCL12 or C–X–C chemokine receptor type 4. The study revealed unprecedented paracrine interactions between EC cells and EMSCs that resulted in the enhancement of transformation phenotypes. Thus, the blocking of TGF-β or CXCL12 signaling can be a therapeutic target for EC.

## INTRODUCTION

Endometrial carcinoma (EC) is one of the leading causes of cancer death in women worldwide [1]. Endometrioid-type EC accounts for 80%–90% of ECs, most of which are estrogen dependent [2]. The delineation of a common mediator of multiple signaling pathways that stimulate the growth and invasiveness of EC cells is warranted to catalyze the development of novel targeted approaches for improving diagnosis and therapy.

Designated for cyclic shedding, the endometrial glandular stroma is rich in endometrial mesenchymal stem cells (EMSCs) that can regenerate both stroma and endometrium of the uterus monthly [3–5]. A study in rodent endometrial stem and progenitor cells revealed their roles in endometrial repair and regeneration, likely through epithelial–mesenchymal transition (EMT) [6]. EMSCs, similar to other mesenchymal stem cells (MSCs), may also play crucial roles in the development of EC [7–11]. However, the mechanism underlying the MSC–carcinoma interaction is unknown.

Interactions between cancers and their adjacent microenvironment play critical roles in the development of cancers, including uterine EC [7–12]. To date, studies have focused on sex hormone signaling and interleukin-6 [13, 14] in stromal cells, but not EMSCs.

By binding to the C–X–C chemokine receptor type 4 (CXCR4), C–X–C ligand (CXCL) 12 is known to be involved in tumor development and metastasis [15–19]. In EC, CXCL12 and CXCR4 were found to be expressed in normal mucosa and EC cells, respectively, and treatment with a neutralizing anti-CXCR4 monoclonal antibody (Ab) reduced metastasis in an EC cell xenograft model [20]. Nevertheless, the involvement of CXCL12 and CXCR4 in the EMSC–EC crosstalk is unknown.

By acting on transforming growth factor (TGF)-β1 receptors (TGFBRs), TGF-β1plays a key role in controlling cell proliferation, differentiation, migration, and apoptosis [21, 22]. TGF-β1 also plays a complex role in tumor biology because it can act as both tumor suppressor and promoter [23, 24]. Notably, TGF-β1 can alternatively inhibit or enhance the growth of malignant phenotypes in many human cancers [25]; indeed, cancer cells often secrete excess TGF-β1 and respond to it through enhanced invasion and metastasis [21]. The expression of TGF-β1 is higher in EC cells than in normal endometrial cells [26].

We proposed that EC cells secrete TGF-β1 to act on TGFBRs on EMSCs and induce CXCL12 expression, which alters the malignant phenotype of EC cells. In the current study, we isolated and characterized human EMSCs and analyzed the crosstalk between EMSCs and EC cells specifically via the TGF-β1/32+252.47R and CXCL12/CXCR4 pathways. The transformation phenotypes, namely cell proliferation, migration and invasion, EMT, and xenograft tumorigenesis, were examined.

## RESULTS

### Isolation and characterization of EMSCs

**Figure 1:**
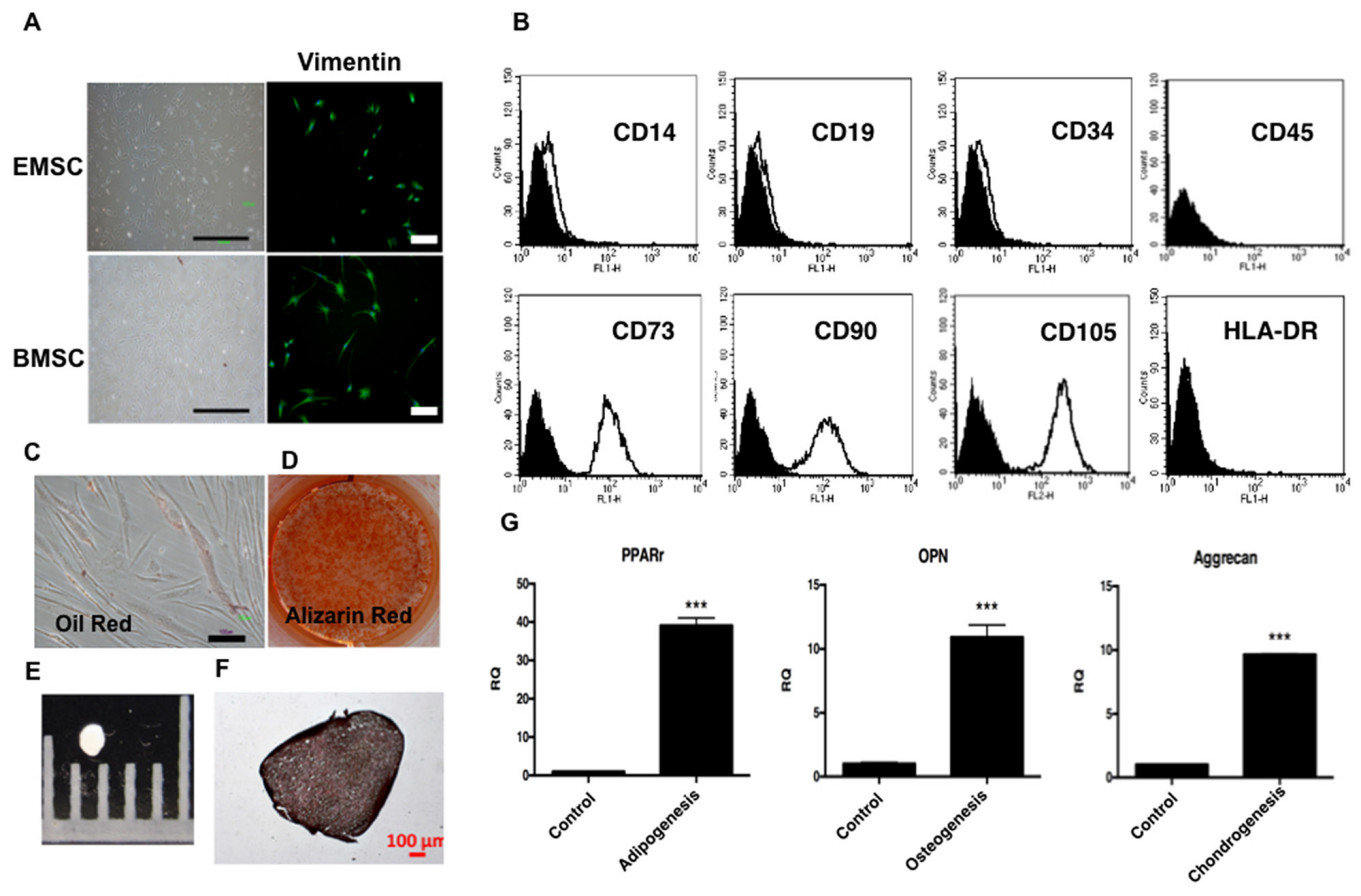
Characterization of EMSCs **(A)** EMSCs with a fibroblast-like morphology and expressed vimentin. Scale bar = 1000 μm in the left panel and 100 μm the in right panel. **(B)** Flow cytometry of EMSCs reveals the expression of CD73, CD90, and CD105, but not of CD14, CD19, CD34, CD45, or HLA-DR. The adipogenic and osteogenic differentiation capability was demonstrated through staining with **(C)** Oil Red and **(D)** Alizarin Red after 2 weeks of differentiation. The chondrogenesis of EMSCs shows **(E)** pellet formation and **(F)** positive staining of type 2 collagen after 3 weeks of differentiation. Scale bar = 100 μm. **(G)** qPCR shows the expression of RNA of *PPAR*-*γ* (adipogenesis), *OPN* (osteogenesis), and *aggrecan* (chondrogenesis). ^***^p < 0.001.

To characterize EMSCs, cell morphology, immunocytochemistry, and surface marker expression and differentiation were examined. The cultured EMSCs (n = 3), similar to bone marrow stromal cells (BMSCs), formed an adherent monolayer with a fibroblast-like morphology in 4–5 days and expressed the mesoderm marker vimentin, as observed by an immunohistochemical (IHC) analysis (Figure 1A). EMSCs expressed typical MSC surface markers, namely CD73, CD90 and CD105. Conversely, known hematopoietic stem cells (HSCs); neural stem cells (NSCs); and endothelial cell markers, namely CD14, CD19, CD34, CD45, and human leukocyte antigen—antigen D related markers (HLA-DR), were not expressed (Figure 1B).

We further analyzed the differentiation, adipogenesis, osteogenesis, and chondrogenesis ability of the EMSCs. After induction for adipogenic+ differentiation, the EMSCs formed Oil Red-positive oil droplets in the cytoplasm (Figure 1C) and expressed adipocyte-specific peroxisome proliferator-activated receptor gamma (*PPAR-*γ) (Figure 1G). Under osteogenic induction, the EMSCs formed an Alizarin Red-positive matrix and expressed the osteopontin (OPN) gene, indicating osteogenic differentiation (Figures 1D and G). After chondrogenesis, pellets were formed (Figure 1E), and an IHC analysis revealed positive staining of type 2 collagen (Figure 1F). Additionally, quantitative polymerase chain reaction (qPCR) showed the gene expression of aggrecan after chondrogenesis (Figure 1G). These results fulfilled the minimal criteria for MSCs [27]. We then obtained three EMSC lines.

### TGF-β1 in normal, hyperplasic, and malignant endometrial tissues and three EC lines, and TGFBRs in EMSCs

**Figure 2:**
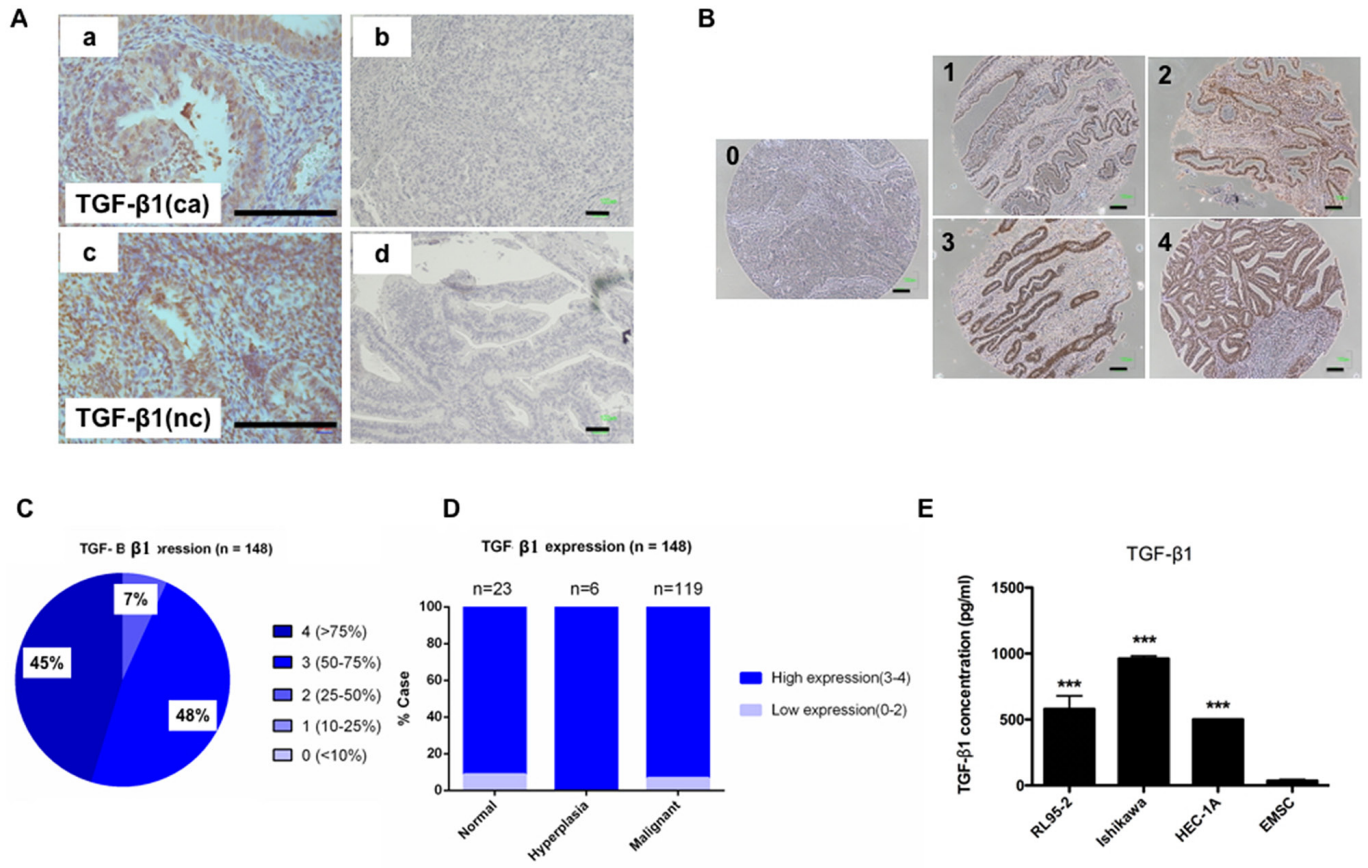
Expression of TGF-β1 in normal endometrial and EC cells **(A)** Representative IHC staining with TGF-β1 Ab (a and c) and immunoglobulin G (IgG)-negative control (b and d) in EC tissues (a and b) and normal endometrial tissues (c and d). **(B)** Representative IHC staining of TGF-β1 in endometrial tissue array (n = 143) with a semiquantitation score of 0–4 based on the percentage of positive cells (brown). Scale bar = 100 μm. **(C)** Distribution of TGF-β1 expression score in 148 cases of ECs. **(D)** Comparison of the TGF-β1 expression in normal, hyperplasia, and malignant cases. (E) ELISA of TGF-β1 in the CM of RL95-2 (579 ± 100 pg/mL), Ishikawa (961 ± 50 pg/mL), and HEC-1A (500 ± 3 pg/mL) cells, and the EMSCs (36 ± 10 pg/mL). ^***^p < 0.001.

To investigate the crosstalk between EC cells and EMSCs, we first investigated the expression of TGF-β1 in endometrial tissues, stem cell (SC) cell lines, and EMSCs through an IHC analysis and enzyme-linked immunosorbent assay (ELISA). Based on a semiquantitative score of IHC staining, TGF-β1 was observed to be equally expressed in normal, hyperplasic, and malignant endometrial tissues in regular block (n = 3) and tissue (n = 148) arrays (Figures 2A–2D). High levels of TGF-β1 were also detected in the conditioned medium (CM) of three EC cell lines: RL95-2 cells (579 ± 100 pg/mL), Ishikawa cells (961 ± 50 pg/mL), and HEC-1A cells (500 ± 3 pg/mL), but not in the CM of EMSCs (36 ± 10 pg/mL; Figure 2E, p < 0.001).

**Figure 3:**
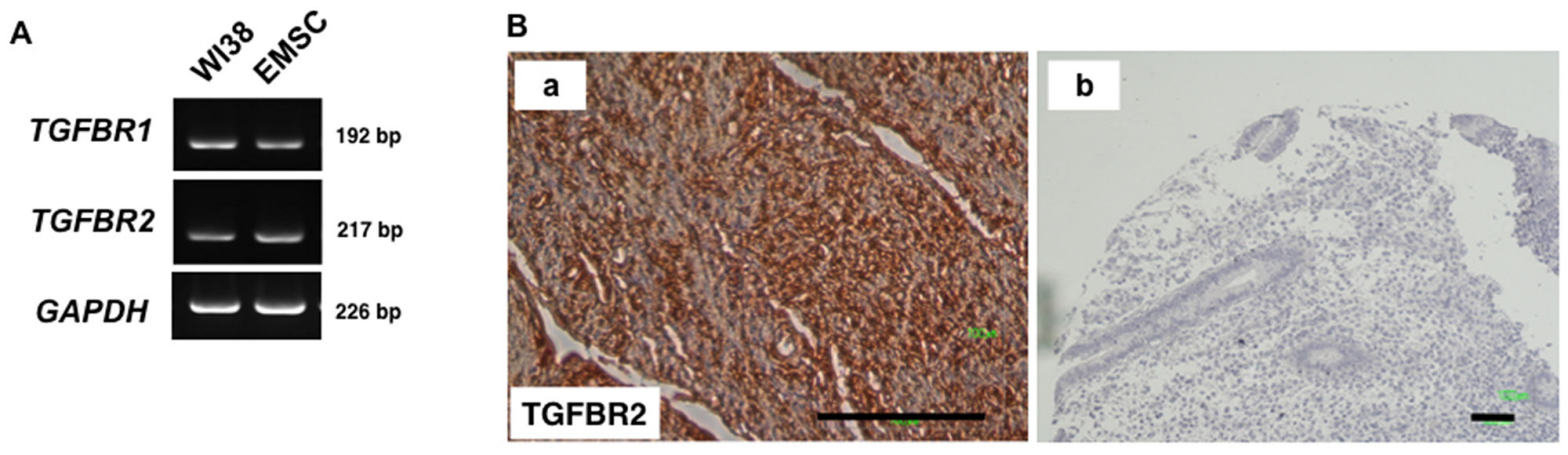
TGFBR2 expression in normal tissues **(A)** RT-PCR analysis reveals the expression of *TGFBR1* and *TGFBR2* in WI38 cells (fetal lung fibroblasts) and the EMSCs (left panel). **(B)** (a)Normal stroma tissues and (b) IgG-negative control of the IHC of *TGFBR2*. Scale bar = 100 μm.

*TGFBR1* and *TGFBR2* messenger ribonucleic acids (mRNAs) were detected in EMSCs (Figure 3A), and the TGFBR2 protein was abundantly expressed in normal endometrial tissues (Figure 3B). These data suggested that TGF-β1 is secreted by normal endometrial and EC cells, and its receptors are expressed in the endometrial stroma and EMSCs.

### RL95-2 CM induces CXCL12 expression in EMSCs through TGFR2

**Figure 4:**
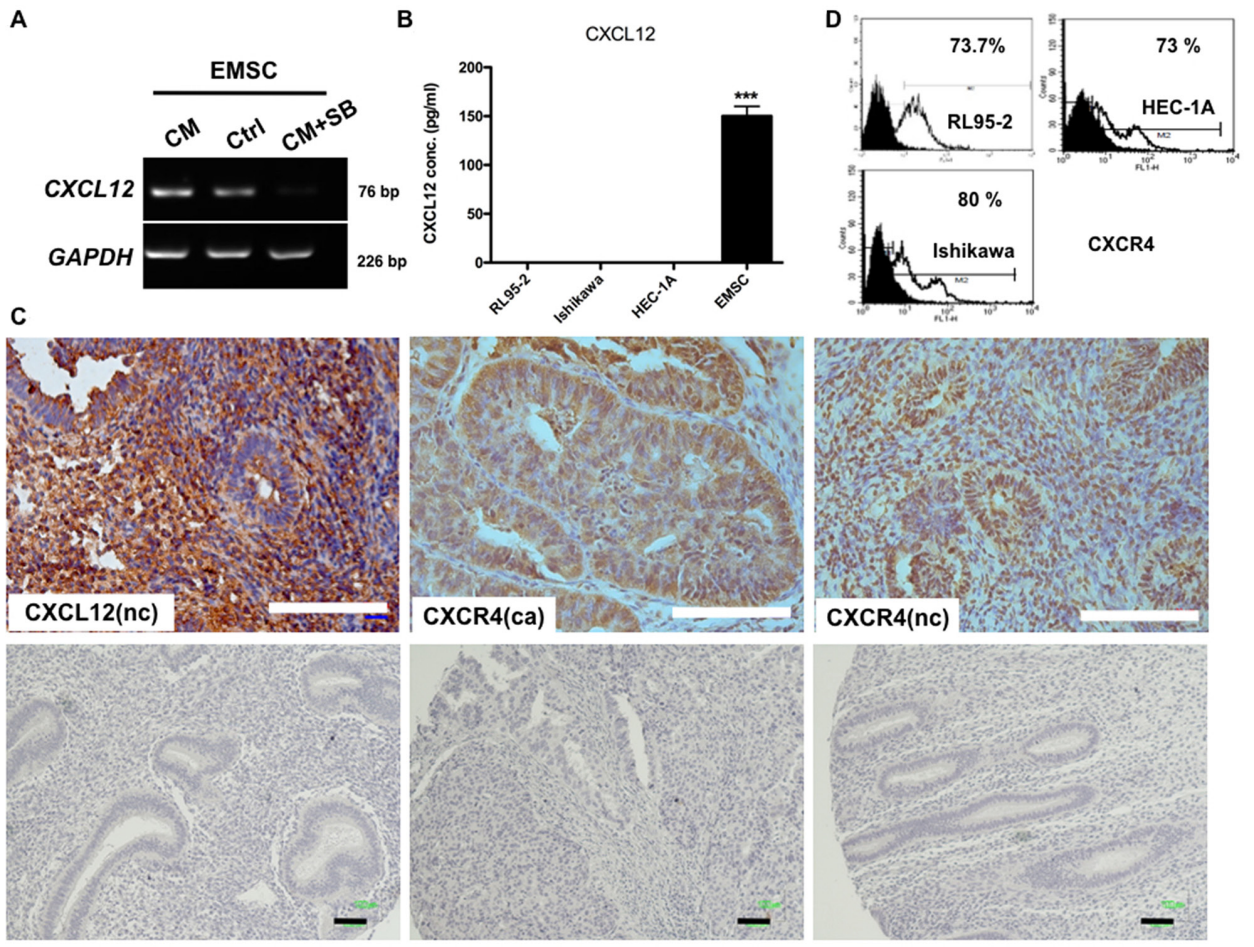
RL95-2 CM and *TGFBR2*-dependent induction of CXCL12 expression in EMSCs, and the normal endometrium and EC expression of CXCR4 **(A)** mRNA of *CXCL12* in the EMSCs measured through RT-PCR. **(B)** ELISA shows the CXCL12 protein in the CM of the EMSCs, but not in the three EC cell lines. **(C)** IHC of CXCL12 and CXCR4 in the normal endometrial cells (nc) and EC cells (ca). IgG-negative control is shown in the lower panel of each figure. Scale bar = 100 μm. ^***^p < 0.001. **(D)** Flow cytometry of CXCR4 on RL95-2, HEC-1A, and Ishikawa cells.

The effects of RL95-2-secreted TGF-β on EMSCs was examined. After treatment with the CM of RL95-2 for 48 h, the expression of *CXCL12* mRNA in EMSCs increased (Figure 4A). Notably, this induction could be blocked by pretreatment with the TGFBR inhibitor SB431542 (Figure 4A). As a confirmation, a high level of CXCL12 was detected in the CM of EMSCs (150 ± 10 pg/mL), but not in that of the RL95-2, Ishikawa, or HEC-1A cells (Figure 4B) or the stroma of normal endometrial cells (Figure 4C). Thus, by binding to its receptor, the TGF-β1 secreted by RL95-2 cells can be inferred to induce the expression of CXCL12 in EMSCs.

### CXCR4 expression in normal endometrial and EC cells

We demonstrated that CXCR4, the receptor of CXCL12, was highly expressed in EC and normal endometrial cells (Figure 4C). Specifically, flow cytometry revealed that most RL95-2 (73.7%), Ishikawa (80%), and HEC-1A (73%) cells expressed CXCR4 (Figure 4D). Thus, the CXCR4-expressing EC cells may readily respond to EMSC-secreted CXCL12.

### EMSC-derived CXCL12 enhances the migration and invasion of EC cells through CXCR4 with increased expression of EMT markers

**Figure 5:**
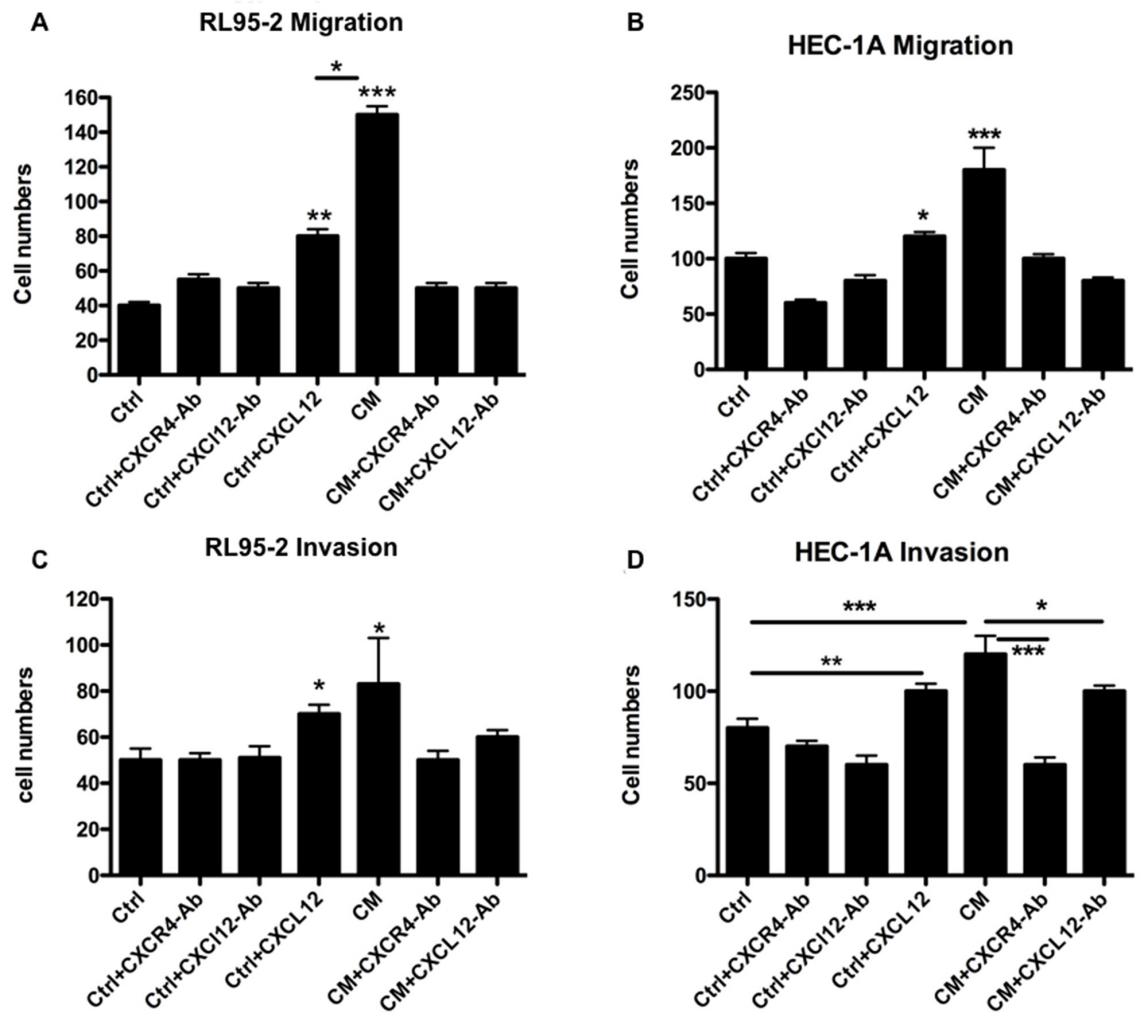
CXCL12 in the CM of the EMSCs acting on the CXCR4 of RL95-2 and HEC-1A cells to enhance the migration and invasion phenotypes Boyden chamber migration and invasion assays of RL95-2 **(A** and **C)** and HEC-1A **(B** and **D)** cells pretreated for 18 h with the CM or Ctrl of the EMSCs with or without 30-min pretreatment with the blocking Ab of CXCR4 (5 μg/mL) or CXCL12 (3 μg/mL). The experiments were conducted in triplicate. The results are expressed as mean ± SEM. ^*^p < 0.05, ^**^p < 0.01, ^***^p < 0.001.

We further examined the consequences of the CXCL12/CXCR4-mediated crosstalk between EMSCs and EC cells. In transwell migration and matrigel invasion assays, the CM of EMSCs significantly promoted the migration (Figures 5A and 5B; p < 0.001) and invasion (Figures 5C and 5D; p < 0.05) of RL95-2 and HEC-1A cells compared with the control medium (Ctrl). However, these increases in transformation phenotypes were readily blocked by treatment with a neutralizing Ab specific to either CXCL12 or CXCR4 (p < 0.001 and p < 0.05, respectively; Figures 5A and 5B).

**Figure 6:**
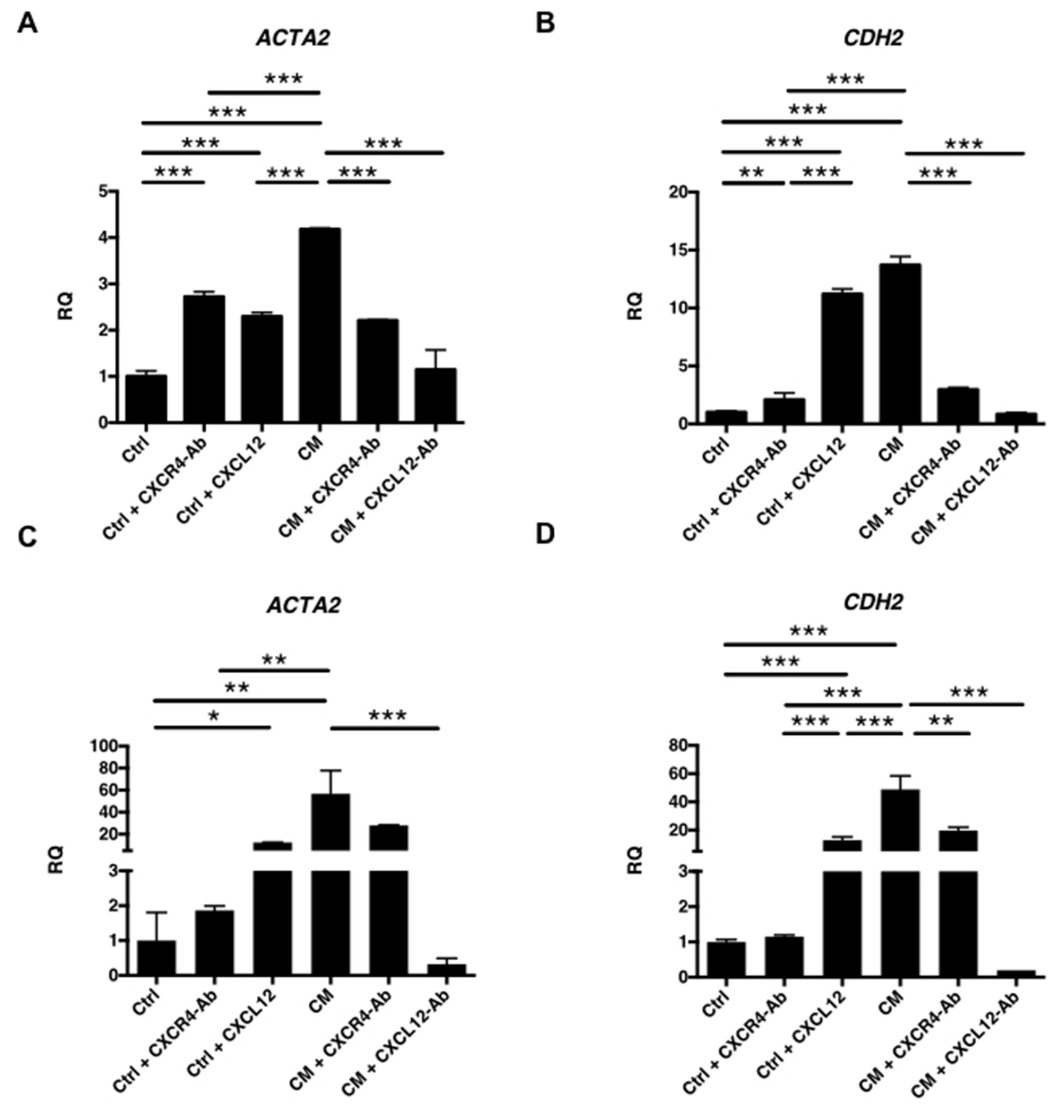
Induction of EMT of EC cells by the EMSCs via CXCL12 and CXCR4 paracrine signaling RT-qPCR analysis of EMT-related genes *ACTA2* **(A and C)** and *CDH2* **(B and D)** in RL95-2 cells (A and B) and HEC-1A cells (C and D) after treatment with the CM or Ctrl of the EMSCs with or without blocking Abs of the blocking Ab of CXCR4 or CXCL12 or Ctrl with CXCL12 protein. The experiments were conducted in triplicate. ^*^p < 0.05, ^**^p < 0.01, ^***^p < 0.001.

Real-time reverse transcription (RT) qPCR was subsequently performed to determine the expression of EMT markers in RL95-2 (Figures 6A and 6B) and HEC-1A (Figures 6C and 6D) cells. Notably, the expression of *ACTA2* (alpha-smooth muscle actin [α-SMA]; Figures 6A and 6C) and *CDH2* (N-cadherin; Figures 6B and 6D) in RL95-2 and HEC-1A cells was increased after treatment with EMSC-CM or pure CXCL12 protein, both of which could be blocked by a neutralizing Ab specific to either CXCL12 or CXCR4. The data suggest that CXCL12 in the CM of EMSCs can enhance the transformation phenotypes of RL95-2 and HEC-1A cancer cells through the CXCR4 receptor and induce an EMT phenotype.

### EMSCs promote the proliferation and xenograft tumor growth of EC cells through the CXCR4 signaling pathway

**Figure 7:**
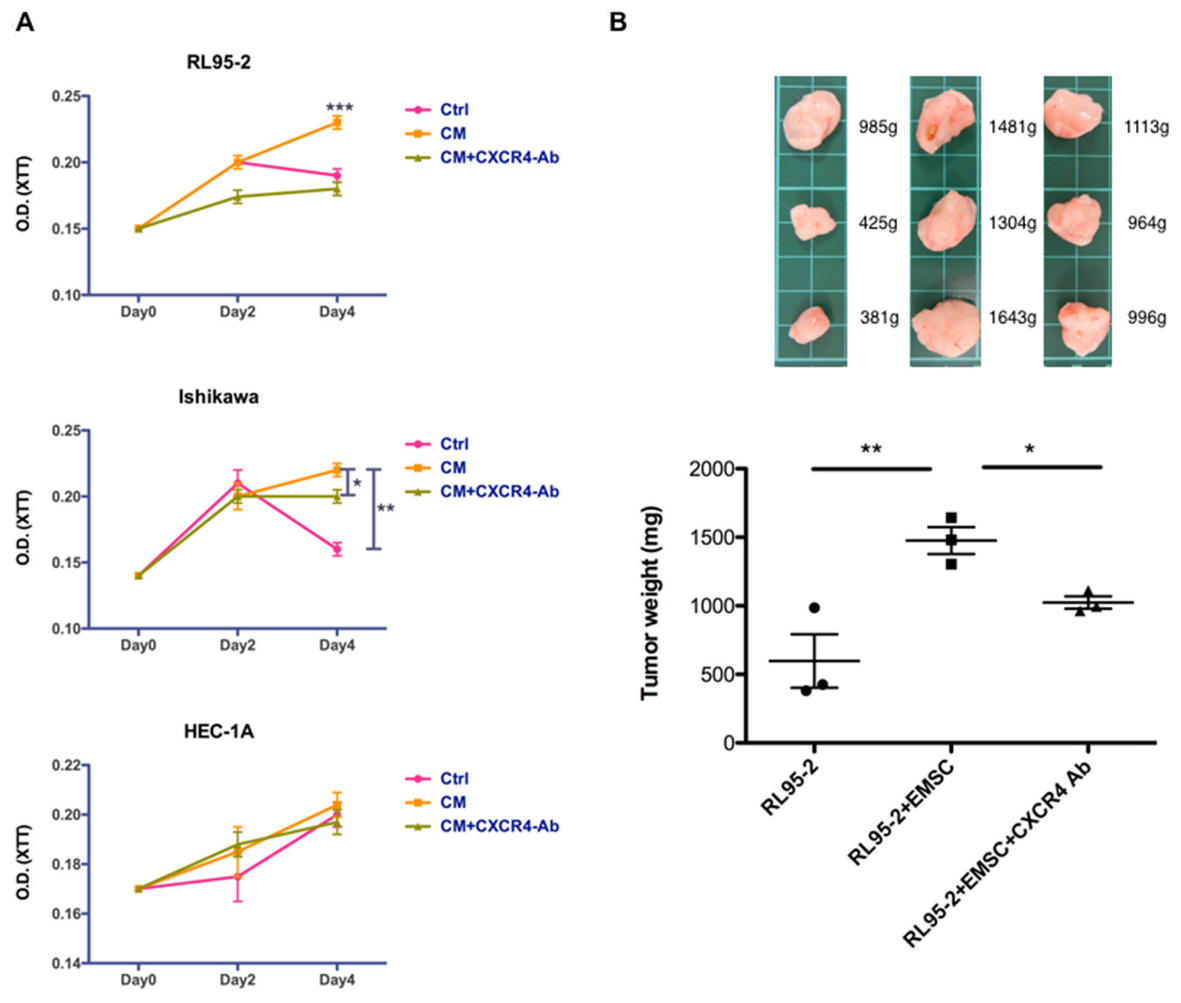
Enhanced proliferation and tumorigenesis of RL95-2 cells via the EMSCs **(A)** RL95-2, Ishikawa, and HEC-1A cells grown in 1:1 mix of EMSC-CM and self culture medium-Ctrl, with or without the anti-CXCR4 Ab (5 μg/mL), or with the Ctrl subjected to the XTT assay at day 0, 2, and 4. The experiments were conducted in triplicate. Data are expressed as mean ± SD. **(B)** RL95-2 cells (5 × 10^5^) alone or in combination with the EMSCs (1.5 × 10^6^cells) s.c. injected or coinjected with the anti-CXCR4 Ab (0.5 mg/kg weight) three times weekly. Data are presented as mean ± SEM. The xenograft tumor morphologies and weights of each group are shown in the upper panel. ^*^p < 0.05, ^**^p < 0.01, ^***^p < 0.001.

The oncogenic effects of the EMSC–EC crosstalk as well as the proliferation of RL95-2, Ishikawa, and HEC-1A cells after treatment with the CM of EMSCs were analyzed using the 2,3-bis(2-methoxy-4-nitro-5-sulfophenyl)-2H-tetrazolium-5 -carboxanilide (XTT) assay. Increased cell proliferation (p < 0.001 in RL95-2 cells and p < 0.01 in Ishikawa cells) was observed following the CM treatment, an effect that was also prevented by pretreatment with the CXCR4 Ab (Figure 7A). In the xenograft tumorigenesis model, a significant increase was observed in tumor weights when RL95-2 cells were coinjected with EMSCs (1476 ± 97 mg) at a 12-week interval, compared with an injection of RL95-2 cells alone (597 ± 194 mg, p < 0.01). Moreover, when the CXCR4 blocking Ab was injected into the RL95-2 + EMSC group, a significant decrease was observed in tumor weights (1025 ± 45 mg, p < 0.05; Figure 7B). Altogether, these results suggest that EMSCs enhanced the growth of RL95-2 and Ishikawa cells *in vitro* and RL95-2 cells *in vivo* via the CXCL12/CXCR4 signaling pathway.

## DISCUSSION

**Figure 8:**
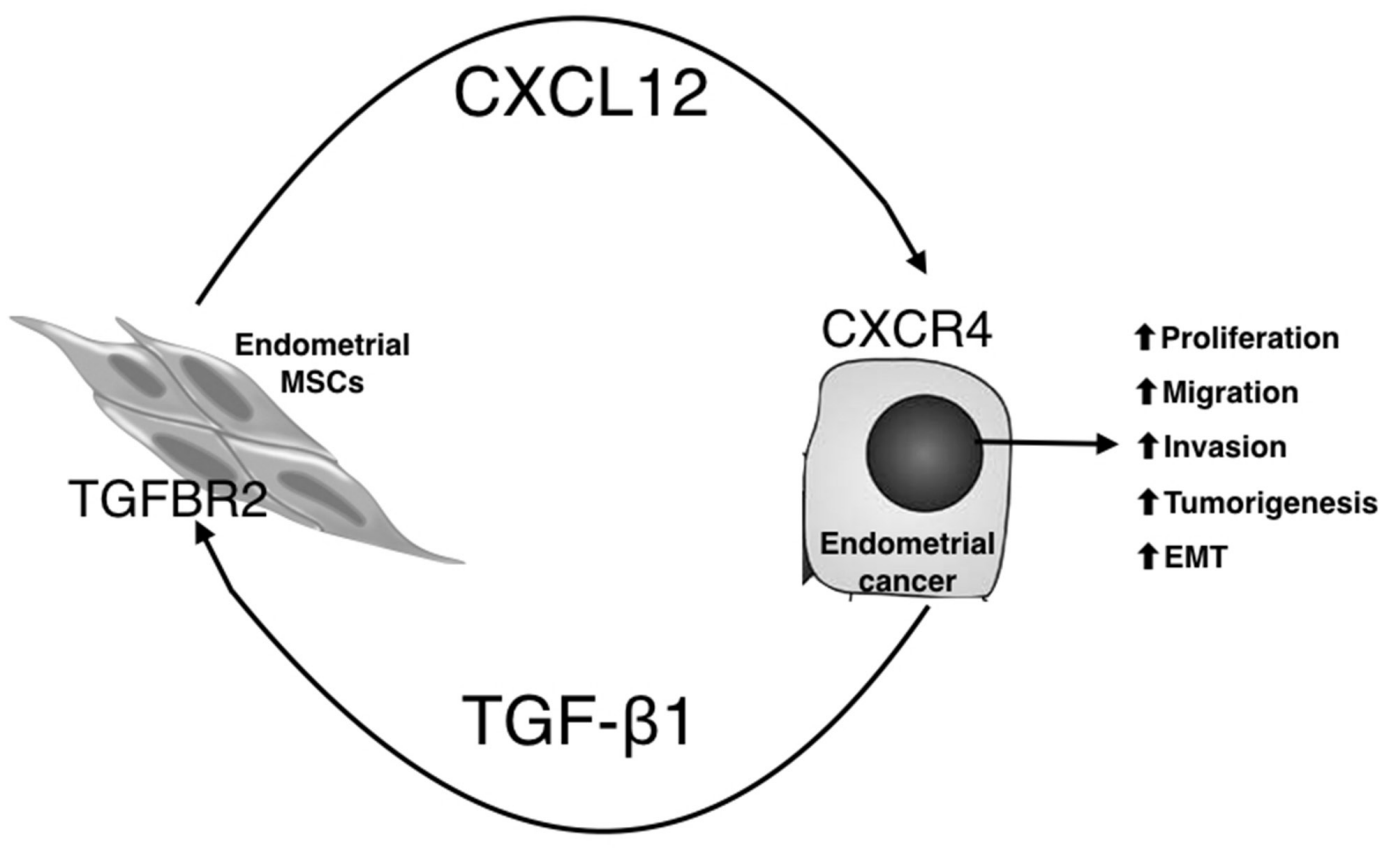
Proposed model for the tumor-promoting effects of EC/EMSC crosstalk through paracrine signaling with TGF-β1, CXCL12, and their receptors EC cells secrete TGF-β1, through TGFR2, to induce EMSCs to secret CXCL12, which by acting on CXCR4 on EC cells, promotes EMT; cell migration, invasion, and proliferation; and tumorigenesis of EC cells.

In this study, EMSCs were successfully cultured from the human endometrium and fulfilled the criteria of MSCs. Reciprocal paracrine interactions were observed between the EC cells and EMSCs, with enhanced transformation phenotypes (Figure 8). Additionally, TGF-β1 was expressed in the EC tissues and cells, whereas its receptors were expressed in the EMSCs. Upon coculturing with EMSCs, the EC cells secreted TGF-β1, which induced CXCL12 secretion from the EMSCs by binding to TGFBRs. Overall, the mutual feedforward and signaling through TGF-β and CXCL12 is associated with the enhancement of EC cell transformation.

The human endometrium is a dynamic remodeling tissue, which undergoes more than 400 cycles of regeneration, differentiation, and shedding during a woman’s reproductive years [3, 28]. After each menstruation cycle, the upper stratum functionalis of the bilayer endometrium structure collectively sheds, leaving the remaining stratum basalis to regenerate in the subsequent cycle [28–30]. The stratum basalis is a mesenchymal tissue rich in EMSCs for regeneration. Present in the basalis of the endometrium, EMSCs are the origin of both epithelial and stromal cells of the new endometrium. As revealed in the present study, EMSCs may also play a crucial role in EC progression.

TGF-β has a complex role in cancer, and it can be involved in cell proliferation, differentiation, migration, and apoptosis [21, 22]. It may act as a tumor suppressor at the initial stage of cancer, but as a stimulator at the advanced stage [25] by contributing to aggressive progression through EMT [31]. In EC, TGF-β signaling supports cell survival and metastasis [32, 33]. Overall, an increase in TGF-β expression correlates with decreased survival and poor prognosis [26, 34].

In the current study, TGF-β1 was expressed in the EC tissues, was secreted into the culture medium of the EC cells, and acted on its receptors on the EMSCs and endometrial stromal tissues. These observations are in accordance with a prior study reporting that TGFBR2 activation in fibroblasts can modulate the oncogenic potential of adjacent epithelia through paracrine hepatocyte growth factor signaling in prostate and stomach tissues [35].

Reciprocally, the EMSCs exposed to the CM of EC cells secreted CXCL12, which acts through CXCR4 in EC cells to promote cell proliferation, migration, invasion, EMT, and tumorigenesis. CXCL12/CXCR4 is known to regulate the critical steps of cancer invasion and metastasis [36]; therefore, EC cells with increased CXCR4 expression have a high metastatic ability [20]. In one previous study, the inhibition of CXCR4 reduced the proliferation and invasion of EC cells and was demonstrated to be an effective therapeutic target [36]. By using Abs to block either the receptor or ligand, the present study confirmed this therapeutic approach and provided a mechanistic basis emphasizing the breakage of the reciprocal feedforward loop between cancer cells and adjacent EMSCs.

## CONCLUSION

The present findings suggest an unprecedented interaction between EC cells and EMSCs through the mutual activation of TGF-β1/TGFBR2 and CXCL12/CXCR4 signaling pathways, which results in enhanced malignant behaviors. This positive feedback loop provides novel therapeutic opportunities for cancer control.

## MATERIALS AND METHODS

### Clinical specimens and cell culturing

This study was approved by the Research Ethics Committee of Tzu Chi General Hospital (IRB 097-111). Informed consent was obtained from all patients and the study protocols were conducted in accordance with relevant guidelines, including all relevant details. Human EMSCs were derived from three patients (aged 40, 45, and 46 years) undergoing hysterectomy for benign tumors. Tumor tissues from another three patients (aged 50, 52, and 53 years) with grade 1 endometrioid adenocarcinoma were procured during surgical staging. All tissue samples were separated into three parts: the first part was fixed in 10% formalin and embedded in paraffin, the second part was suspended in a RNA solution for RT-PCR analysis, and the third part was sent for primary cell culturing.

### EMSC derivation and characterization

The detailed method of EMSC culturing is described elsewhere [37]. Briefly, the collected basalis human endometrial tissues were washed three times with Ca^2+^ and Mg^2+^-free phosphate-buffered saline (PBS, Life Technology, Carlsbad, CA, USA). The specimen was cut into pieces smaller than 0.5 cm^3^, treated with collagenase type 1 (Sigma, St Louis, USA), and incubated for 14–18 h at 37°C in a 95% air and 5% CO_2_-humidified atmosphere. The explants were then cultured in Dulbecco’s modified Eagle medium (DMEM) containing 10% fetal bovine serum (FBS) and antibiotics at 37°C in a 95% air and 5% CO_2_-humidified atmosphere. They were left undisturbed for 5–7 days to allow for the migration of cells from the explants.

### Flow cytometric analysis

EMSCs were characterized through fluorescence-activated cell sorting (FACS) with cytometric markers, namely CD73, CD90, and CD105 (positive markers) and CD14, CD19, CD34, CD45, and HLA-DR (negative markers) [27]. EMSCs between the third and eighth passages were used for analysis.

FACS was also performed to quantify the cell surface or intracellular expression of CXCR4 in RL95-2 cells. Briefly, trypsinized cell pellets were fixed with 0.4% formaldehyde and PBS and lysed using 90% ice-cold methanol. After the cells were washed three times with PBS, they were incubated with a green fluorescent protein-conjugated monoclonal Ab, specific for CXCR4 (R&D), for 30 min at 4°C; subsequently, the cells were rewashed three times with PBS. FACS data were acquired on a FACS Caliber (Becton Dickinson, San Jose, CA, USA) and analyzed using CELL Quest software (Becton Dickinson, Franklin Lakes, NJ, USA).

### *In vitro* differentiation assay for EMSCs

A detailed discussion of *in vitro* differentiation is reported in a previous study [37]. Briefly, passage 2–3 of EMSCs was seeded in a 12-well plate at a density of 5 × 10^4^ with an adipogenic medium (DMEM supplemented with 10% FBS, 1 μmol/L dexamethasone, 5 μg/mL insulin, 0.5 mmol/L isobutylmethylxanthine, and 60 μmol/L indomethacin). These cells were grown for 14 days, and the medium was changed every 3 days. The passage 2–3 of EMSCs was then seeded in a 12-well plate at a density of 1 × 10^4^ and grown with an osteogenic medium (DMEM supplemented with 10% FBS, 0.1 μmol/L dexamethasone, 10 mmol/L β-glycerol phosphate, and 50 μmol/L ascorbate), which was changed every 3 days. These cells were grown for 21 days.

The osteogenic differentiation potential was assessed through Alizarin Red S staining to determine calcium mineralization. For assessing adipogenic differentiation, intracellular lipid droplets were observed through microscopy and confirmed through Oil Red O staining. Then, for inducing chondrogenesis, EMSCs were seeded in a 15-mL conical tube at a density of 1 × 10^6^ cells/cm^2^ and grown in a chondrogenic medium (DMEM containing 10% FBS, 10 ng/mL TGF-β1 [Pepro Tech Inc., Rocky Hill, NJ, USA], 50 μg/mL ascorbic acid-2-phospate [Sigma–Aldrich], and 6.25 μg/mL of insulin [Sigma–Aldrich]). The medium was changed every 3 days, and the cells were incubated with a chondrogenic medium at 37°C with 5% CO_2_ for 3 weeks. After being fixed in paraformaldehyde (Bionovas, Toronto, Canada), the resultant pellets were sliced into 5-μm-thick pieces and mounted on slides, followed by IHC staining with type 2 collagen (Sigma–Aldrich).

### Reagents

CXCR4, CXCL12, and SB431542 were purchased from Sigma (St. Louis, MO, USA) and the neutralizing Abs for CXCR4 and CXCL12 were purchased from R&D Systems Inc. (Minneapolis, MN, USA). To study TGF-β signaling, a small molecular inhibitor (SB431542, Sigma) was used.

### Immunohistochemistry

Four-micrometer-thick paraffin sections were treated with 0.01 M citrate buffer at 90°C and heated for 5 min in a microwave oven at 750 W. IHC staining for CXCR4 and TGF-β1 was performed using the avidin–biotin immunoperoxidase technique (Histofine SAB-PO kit, Nichirei, Tokyo, Japan), according to the manufacturer’s instructions. Anti-CXCR4 and anti-TGF-β1 polyclonal Ab (Chemicon, Temecula, CA, USA) were used at a dilution of 1:100.

Vimentin (dilution 1:200; Chemicon) was used to identify isolated cells originating from the mesenchyme. The cells were fixed in 4% paraformaldehyde and permeabilized with PBS containing 5% skim milk (Becton Dickinson) and 0.1% Triton X-100 for 30 min. The cells were subsequently incubated with mouse antihuman monoclonal Abs overnight. After washing with PBS containing 0.5% Tween 20, the cells were incubated with fluorescein isothiocyanate-conjugated secondary Abs for 30 min. Negative and positive control slides were prepared by incubating the sections with isotype controls instead of primary Abs. Nuclei were counterstained with Hoechst 33342. The cells were then washed three times with PBS and observed through fluorescence microscopy (Olympus, Tokyo, Japan).

### Tissue microarray

A tissue microarray containing five normal and benign and 70 primary EC (Federation of Gynecology and Obstetrics stage 1–4) tissues, was purchased from Abcam (ab178150). The IHC staining of TGF-β1 was performed using a steady DAB/Plus (Abcam) kit. The stained cores were evaluated by two independent observers and scored according to the percentage of immunopositive tumor cells: 0 = <10%, 1 = 1%–25%, 2 = 25%–50%, 3 = 30%–75%, and 4 = >75% [38].

### Cancer cell lines

RL95-2, HEC-1A, and Ishikawa cell lines were purchased from the Food Industry Research and Development Institute of Taiwan (Hsinchu, Taiwan). RL95-2 cells were obtained from Caucasians, and the remaining cell lines were derived from Asians. The culture medium for RL95-2 consisted of 90% DMEM:F12 (1:1, Invitrogen, Thermo Fisher Scientific, Waltham, MA, USA) with 10 mM 4-(2-hydroxyethyl)-1-piperazineethanesulfonic acid (Sigma–Aldrich), 5 μg/mL bovine insulin (Sigma–Aldrich), 2 g/mL sodium bicarbonate (Sigma–Aldrich), and 10% FBS (Biological Industries, Kibbutz, Israel). Ishikawa cells were cultured in minimal essential medium (MEM) with 2 mM glutamine, 1% nonessential amino acids (Sigma–Aldrich), and 5% FBS (Biological Industries). Finally, HEC-1A cells were grown in McCoy’s 5a medium modified (ATCC 30-2007, USA) with 10% FBS (Biological Industries).

### Migration assay

RL95-2 and HEC-1A cells were seeded in the upper well of a 24-well transwell Boyden chamber (pore size: 8 μm; Costar) and allowed to migrate toward the cell-free media derived from the EMSCs placed in the bottom wells. After 18 h, the cells that had migrated onto the membrane insert were stained with crystal violet and counted through bright-field microscopy. To evaluate the role of paracrine signaling on this migration, CXCL12 protein (100 ng/mL), CXCL12 (3 μg/mL), and CXCR4 (5 μg/mL) blocking Abs (R&D) were added into the CM.

### Invasion assay

Next, RL95-2 and HEC-1A (5 × 10^4^) cells were seeded in the matrigel-coated inserts of 24 wells for 24 h (BD Biocoat Matrigel Invasion Chamber, BD Bioscience, Bedford, MA, USA) and allowed to invade the cell-free media derived from the EMSCs placed in the bottom wells. Following the manufacturer’s protocol, we removed the membrane and placed it on a slide, and then observed the invading cells through microscopy. The cells were counted in several fields in triplicate. To evaluate the role of paracrine signaling in this invasion, CXCL12 protein (100 ng/mL), CXCL12 (3 μg/mL), and CXCR4 (5 μg/mL) blocking Abs (R&D) were added into the CM.

### ELISA for TGF-β1 and CXCL12

To prepare the CM, RL95-2, HEC-1A, and Ishikawa cells, as well as the EMSCs (5 × 10^5^ cells) were plated on 100-mm cell culturing dishes and cultured in a growth medium for 1 day. The cells were briefly rinsed twice with PBS and then fed with a serum-free α-MEM for 48 h. Next, the CM was centrifuged at 2000 rpm for 10 min to remove cell debris, filtered using 0.45-μm Millipore Ultrafree centrifugal filters (Millipore, Bedford, MA, USA), and stored at −70°C for subsequent use. Finally, the concentrations of CXCL12 and TGF-β1 were determined using ELISA (R&D).

### RT-PCR and qRT-PCR

The total RNA was prepared from the EMSCs and RL95-2 and HEC-1A cells by using Trizol, according to the manufacturer’s instructions (Invitrogen, Carlsbad, CA, USA). A total of 1 μg of DNase-treated RNA was transcribed into complementary deoxyribonucleic acid by using 200 units of Super-Script II reverse transcriptase (Invitrogen) and 150 ng of random primers (Invitrogen). RT-PCR was performed using the Qiagen OneStep RT-PCR kit (Hilden, Germany) under appropriate PCR conditions and with the following primers: WI38 cells were fetal lung fibroblasts used as the control cell line for the RT-PCR of TGFBR1 and TGFBR2, and SB-431542 (Sigma) was used to inhibit the effects of TGF-β1 in the EMSCs. The sequences of candidate genes are listed in Table 1.

**Table 1:** Primer set of various genes used in qPCR and RT-PCR

Gene	Sense (5′-3′)	Antisense (5′-3′)	Product size (bp)
*GAPDH*	GGCAGCAGCAAGCATTCCT	GCCCAACACCCCCAGTCA	226
*PPAR-γ*	AGCCTCATGAAGAGCCTTCCA	TCCGGAAGAAACCCTTGCA	120
*Osteopontin*	AGGAGGAGGCAGAGCACA	CTGGTATGGCAC AGGTGATG	150
*Aggrecan*	CGAAACATCACTGAGGGTGA	GCAAACGTGAAGGGCTCCT	170
*TGFBR1*	TGA ACA GAA GTT AAG GCC AAA TAT C	CAG GCA AAG CTG TAG AAT TAC ATT T	192
*TGFBR2*	CGG TTA ATA ACG ACA TGA TAG TCA C	TCA TGG CAA ACT GTC TCT AGT GTT A	217
*CXCL12*	ATGAACGCCAAGGTCGTGG	CCAGGTACTCCTGAATCCAC	76
*ACTA2*	CCT GAC TGA GCG TGG CTA TT	GAT GAA GGA TGG CTG GAA CA	226
*CDH2*	ACC AGG TTT GGA ATG GGA CA	ACA TGT TGG GTG AAG GGG TG	156

For the quantitative analysis of *PPARγ* (an adiopocyte marker), *OPN* (an osteocyte marker), *aggrecan* (a chondrocyte marker), *ACTA2* (α-smooth muscle actin), and *CDH2* (N-cadherin, an EMT marker), the FastStart Universal SYBR Green Master (ROX, Roche, Indianapolis, IN, USA) was used. Gene expression assays were performed in an ABI StepOnePlus system (Applied Biosystems, Foster City, CA, USA), with glyceraldehyde 3-phosphate dehydrogenase (*GAPDH*) as the internal control. At the end of the reaction, quantification and melting curve analyses were performed according to the manufacturer’s instructions.

### Proliferation assay

The proliferation of RL95-2, Ishikawa, and HEC-1A cells grown in 1:1 mix of EMSC-CM and self culture medium-Ctrl were determined using XTT assays on culturing days 0, 2, and 4. The culture medium was changed every 2 days. Additionally, the blocking assay was used with CXCR4 (5 μg/mL) blocking Abs (R&D). The assays were performed using the XTT reagent mixture (Roche, Germany) in accordance with the manufacturer’s protocol.

### *In vivo* studies

All animal protocols were approved by the Institutional Animal Care and Use Committee at Buddhist Tzu Chi General Hospital. Five-week-old female NOD scid gamma mice (strain: NOD.CB17-Prkdcscid/JTcu) were obtained from the animal center of Tzu Chi University in Taiwan and were divided into three groups (n = 3 in each group). The first group had RL-95-2 cells (5 × 10^5^ cells each/0.5 mL of medium/mouse) injected subcutaneously (s.c) at the back region. The second group was coinjected with RL95-2 cells (5 × 10^5^ cells) and EMSCs (1.5 × 10^6^ cells each/0.5 mL of medium/mouse). The third group was coinjected with RL95-2 cells (5 × 10^5^ cells) and EMSCs (1.5 × 10^6^ cells), and was s.c injected with the CXCR4 blocking Ab (0.5 mg/kg weight) three times a week. Tumorigenesis was examined weekly. The mice were sacrificed 12 weeks after the injection and the tumor occurrence and weights were measured. Notably, the tumors did not exceed 2 cm in diameter.

### Statistical analysis

For data of *in vitro* and *in vivo* experiments, statistical comparisons among the groups were performed using Student t tests and an analysis of variance with Bonferroni corrections. Differences among the groups were considered statistically significant at p < 0.05. All data are expressed as mean ± standard deviation (SD) or standard error of the mean (SEM), as indicated in the results.
